# Double tract reconstruction improves the quality of life and better maintain the BMI of patients with proximal gastric cancer

**DOI:** 10.1186/s12893-024-02454-8

**Published:** 2024-05-31

**Authors:** Zi jian Wang, Zi yao Xu, Zi jie Huang, Li Li, Da Guan, Yun he Gao, Xin xin Wang

**Affiliations:** Department of General Surgery, the First Medical Center of Chinese PLA General Hospital, Beijing, China

**Keywords:** Gastric cancer, Proximal gastrectomy, Double tract reconstruction

## Abstract

**Purpose:**

The aim of this study is to investigate the effect of double-tract reconstruction on short-term clinical outcome, quality of life and nutritional status of patients after proximal gastrectomy by comparing with esophagogastrostomy and total gastrectomy with Roux-en-Y reconstruction.

**Methods:**

The clinical data of patients who underwent double tract reconstruction (DTR), esophagogastrostomy (EG), total gastrectomy with Roux-en-Y reconstruction (TG-RY) were retrospectively collected from May 2020 to May 2022. The clinical characteristics, short-term surgical outcomes, postoperative quality of life and nutritional status were compared among the three groups.

**Results:**

Compared with the DTR group, the operation time in the TG group was significantly shorter (200(180,240) minutes vs. 230(210,255) minutes, *p* < 0.01), and more lymph nodes were removed (28(22, 25) vs. 22(19.31), *p* < 0.01), there were no significant differences in intraoperative blood loss, first flatus time, postoperative hospital stay and postoperative complication rate among the three groups. Postoperative digestive tract angiography was completed in 36 patients in the DTR group, of which 21 (58.3%) showed double-tract type of food passing. The incidence of postoperative reflux symptoms was 9.2% in the DTR group, 43.8% in the EG group and 23.2% in the TG group, repectively (*P* < 0.01). EORTCQLQ-STO22 questionnaire survey showed that compared with EG group, DTR group had fewer reflux symptoms (*P* < 0.05), fewer anxiety symptoms (*P* < 0.05) and more swallowing symptoms (*P* < 0.05). Compared with TG group, DTR group had fewer reflux symptoms (*P* < 0.05). There were no other significant differences between the two groups. Compared with TG group and EG group, DTR can better maintain postoperative BMI, and there is no statistical difference between the three groups in terms of hemoglobin and albumin.

**Conclusions:**

Although partial double-tract reconstruction approach does not always ensure food to enter the distal jejunum along the two pathways as expected, it still shows satisfactory anti-reflux effect. Moreover, it might improve patients’ quality of life and maintain better nutritional status comparing with gastroesophageal anastomosis and total gastrectomy with Roux-en-Y reconstruction.

## Introduction

Gastric cancer ranks the fifth in the global cancer morbidity spectrum and the fourth in the cause of death spectrum [1]. In recent years, the overall incidence of gastric cancer has shown a downward trend But the incidence of proximal gastric cancer has steadily increased [2, 3].

At present, total gastrectomy is the primary surgical procedure for proximal gastric cancer .On the one hand, this is due to the consideration of radical tumor treatment and on the other hand, the incidence of reflux esophagitis caused by Roux-en-Y reconstruction after total gastrectomy is very low. However, after proximal gastrectomy, reflux occurs in about one third of patients [4, 5]. but total gastrectomy significantly impairs the long-term health related quality of life (HRQoL) of the patients.

In recent years, with the wide application of minimally invasive surgical techniques, the concept of ensuring radical resection of tumors while paying attention to the quality of life and nutritional status of patients after surgery is driving the proportion of proximal gastrectomy in the treatment of proximal gastric cancer to increase [6]. some prospective study suggested the safety and radicality of proximal gastrectomy as an alternative to total gastrectomy [7]. Laparoscopic proximal gastrectomy is increasingly preferred for operative management of early gastric cancer,

Esophagogastrostomy and Double tract reconstruction are commonly used in digestive tract reconstruction after proximal gastrectomy .Although there is no consensus on a standard reconstruction method after resection .The double-tract reconstruction has been recognized by surgeons for its good anti-reflux effect [8]. In theory, double-tract reconstruction is an ideal reconstruction method after proximal gastrectomy. Food can enter the distal digestive tract through remnant stomach or jejunum, which not only solves the problem of esophageal reflux after proximal gastrectomy, but also preserves the storage and digestive function of the remnant stomach [9–11]. Most studies show that the anti-reflux effect of double-tract reconstruction is accurate [12, 13]. As for the effectiveness of double-tract reconstruction, some studies have suggested that in some cases food cannot be emptied in accordance with the theoretical double-channel design, and when most food passes directly through the jejunum, its function will be similar to that of total gastrectomy [14]. At present, there are few studies on the quality of life and nutritional status of different digestive tract reconstruction methods after radical proximal gastrectomy, and the conclusions are still controversial.

The aim of the present study is to investigate the effect of double-tract reconstruction by comparing operative outcomes, postoperative nutritional state, among patients treated with double tract reconstruction, esophagogastrostomy, and total gastrectomy with Roux-en-Y reconstruction. In addition, to investigate the effect of two states of single channel and double channel actually presented after double tract reconstruction on patients. Whether there is a difference in function between double-tract reconstruction and total gastrectomy when food is emptied only along a single tract .

## Materials and methods

### Patients

The clinical data of patients who underwent radical gastrectomy for proximal gastric cancer at Department of General Surgery, the First Medical Center of Chinese People’s Liberation Army General Hospital from June 2020 to June 2021 were retrospectively analyzed. A total of 295 patients were included in this study. Patients were classified retrospectively based on the reconstructive procedure into an EG group (*n* = 96), DTR group (*n* = 87), and TG group (*n* = 112).

### Surgical procedure

Five working ports were inserted into the umbilicus (12 mm), right upper quadrant(5 mm), right lower quadrant (12 mm), left upper quadrant(5 mm), and left lower quadrant (5 mm). In the laparoscopic proximal gastrectomy, D1 + lymph node dissection was performed including lymph node stations 1,2,3, 4sa, 4sb, 7, 8a, 9, and 11p. In the laparoscopic total gastrectomy, D2 lymph node dissection was performed including lymph node stations 1,2,3, 4sa, 4sb, 4d,5,6,7, 8a, 9, 11p,11d and 12a.The digestive tract was reconstructed as follows:

1. Esophagogastrostomy: End-to-side anastomosis was performed between the esophagus and the remnant stomach with a circular stapler, and the anastomosis was located in the anterior wall of the remnant stomach. anchoring the gastric wall to the diaphragm to create a neo-His angle and fundus. Figure 1 shows the Schematic diagram of Esophagogastrostomy.

**Fig. 1:**
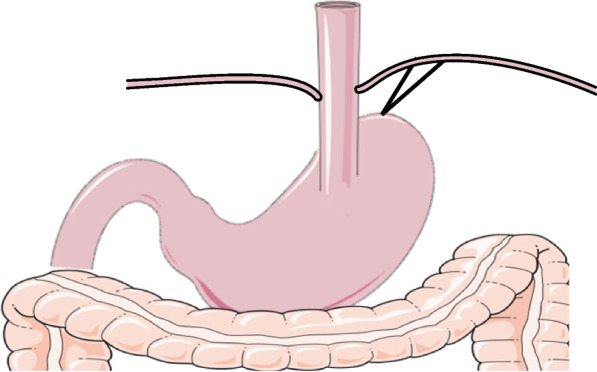
Schematic diagram of Esophagogastrostomy (**a**). Routine dissection of lymph nodes, transection of esophagus and resection of proximal stomach; (**b**). The esophageal stump was placed with a circular stapler against the nail seat, and the esophageal stump was fixed with a purse string; (**c**). The stapler was inserted through the incision of the stomach, and the anterior wall of the stomach was connected with the esophageal nail seat 3 cm away from the top of the remnant stomach, and the end-to-side esophagogastric anastomosis was performed; (**d**). The remnant stomach is fixed at the foot of the diaphragm to reconstruct the artificial “gastric fundus”
2. Double tract reconstruction: The jejunum 25 cm distal to the ligament of Treitz was cut, The distal jejunum was anastomosed end-to-side to the esophagus using a circular stapler, Side-to-side jejunal anastomosis was performed 50 cm away from the esophagojejunal anastomosis, Finally, a 60-mm straight-line cutter was used 15 cm away from the esophagojejunostomy to perform side-to-side anastomosis between jejunum and the anterior wall of the remnant stomach. Figure 2 shows the Schematic diagram of double tract reconstruction.

**Fig. 2:**
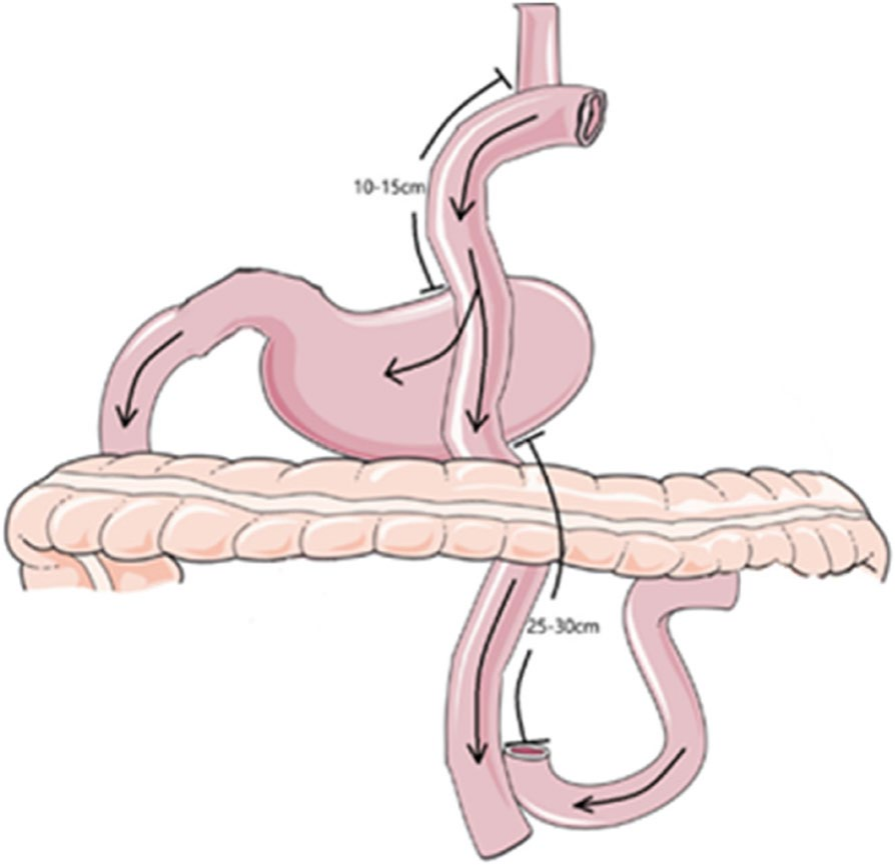
Schematic diagram of Double tract reconstruction (**a**). Routine dissection of lymph nodes, esophagus transection, tumor and proximal stomach resection; (**b**). jejunum and mesenteric vessels were cut off 20 to 25 cm from the suspensory ligament of the duodenum; (**c**). The distal jejunum was anastomosed end-to-side to the esophagus using a circular stapler, Side-to-side jejunal anastomosis was performed 50 cm away from the esophagojejunal anastomosis; (**d**). a 60-mm straight-line cutter was used 15 cm away from the esophagojejunostomy to perform side-to-side anastomosis between jejunum and the anterior wall of the remnant stomach
3. Total gastrectomy with Roux-en-Y reconstruction: The jejunum 25 cm distal to the ligament of Treitz was cut, and the distal jejunum was anastomosed with the esophagus end-to-side with a circular stapler, and the side-to-side anastomosis of the jejunum was performed 50 cm distal to the esophagojejunal anastomosis. Figure 3 shows the Schematic diagram of total gastrectomy with Roux-en-Y reconstruction.

**Fig. 3:**
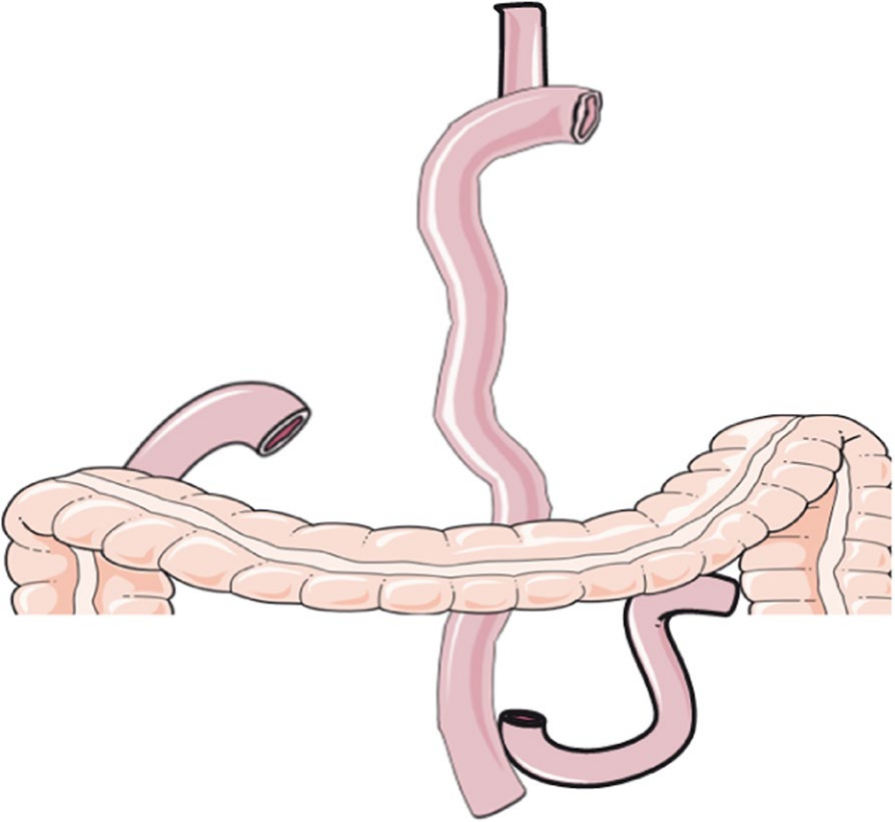
Schematic diagram of total gastrectomy with Roux-en-Y reconstruction (**a**). Routine dissection of lymph nodes, transection of esophagus and resection of total stomach; (**b**). The jejunum 25 cm distal to the ligament of Treitz was cut, and the distal jejunum was anastomosed with the esophagus end-to-side with a circular stapler; (**d**). the side-to-side anastomosis of the jejunum was performed 50 cm distal to the esophagojejunal anastomosis

### Clinical analysis

The clinical and pathological characteristics of patients collected included age, sex, body mass index (BMI), tumor location, degree of differentiation, tumor size, pT category, pN category, pTNM category, number of harvested lymph nodes, The intraoperative and postoperative parameters included postoperative hospital stay, blood loss volume, operation duration, postoperative complications. Complications were defined according to the Clavien-Dindo classification system. The patients were followed up by outpatient examination or telephone at 1 year after surgery. The BMI, nutritional indicators, double-tract reconstruction digestive tract angiography, Investigation of reflux status and EORTC QLQ-STO22 questionnaire were recorded at 1 year after surgery.

### Statistical analysis

SPSS 26.0 statistical software was used to analyze the data. Descriptive analysis was performed on the baseline data. Measurement data with normal distribution were represented as mean ± standard deviation, and measurement data with skewed distribution were represented as median (interquartile range). For categorical variable data, the number (percentage) was used to describe the data. The t-test was used to compare the measurement data that conformed to normal distribution, the chi-square test was used to compare the frequency data, and the rank sum test was used to compare the rank data and the measurement data that did not conform to normal distribution. *P* < 0.05 was considered statistically significant.

## Results

### Clinical and pathological characteristics

A total of 295 patients were enrolled in the present study. Among them, 96 patients underwent esophagogastrostomy reconstruction, 87 patients underwent double tract reconstruction, and 112 patients underwent total gastrectomy with Roux-en-Y reconstruction. Regarding patient demographics, there were no statistically significant differences observed among the three groups in terms of age, sex, body mass index (BMI), and American College of Anesthesiologists (ASA) classification. Furthermore, no significant differences were found among the three groups in terms of pathological features, including tumor differentiation, tumor location, and T stage. Statistical disparities in tumor size were observed among the three groups, namely EG (3: 2.3, 3.8), DTR (2.5: 2, 4), and TG (3.5: 1.8, 4.2) (*p* = 0.03). Furthermore, significant statistical differences were found in the N stage across the three groups. Specifically, the DTR group exhibited an earlier N stage, while the TG group displayed a later N stage (*p* = 0.008). The clinicopathological characteristics of all patients are presented in Table 1.

**Table 1:** Clinicopathological characteristics of the patients

	EG (*n* = 96)	DTR (*n* = 87)	TG + RY (*n* = 112)	*P* value
Mean age (years)	64.8 ± 6.62	63.45 ± 6.67	63.15 ± 9.64	0.296
Sex, n (%)				
Male	74 (77.1)	69 (79.3)	81 (72.3)	0.494
Female	22 (22.9)	18 (20.7)	31 (27.7)	
Body mass index, kg/m2	23.79 ± 2.79	24.24 ± 3.27	24.75 ± 3.36	0.636
ASA				
1	8 (8.3)	12 (13.8)	17 (15.2)	0.265
2	82 (85.4)	64 (73.6)	86 (76.8)	
3	6 (6.3)	11 (12.6)	9 (8.0)	
Differentiation				
Well differentiated	28 (29.2)	18 (20.7)	42 (37.5)	0.055
Moderately differentiated	52 (54.2)	52 (59.8)	60 (53.6)	
Poorly differentiated	16 (16.7)	17 (19.5)	10 (8.9)	
Tumor location				
Upper third	68 (70.8)	56 (64.4)	60 (53.6)	0.082
Upper and middle third	22 (22.9)	23 (26.4)	44 (39.3)	
Middle third	6 (6.3)	8 (9.2)	8 (7.1)	
Tumor size (mm)	3 (2.3, 3.8)	2.5 (2, 4)	3.5 (1.8, 4.2)	0.03
Pathologic T classification				
I	26 (27.1)	30 (34.5)	23 (20.5)	0.174
II	21 (21.9)	22 (25.3)	26 (23.2)	
III	49 (51)	35 (40.2)	63 (56.3)	
Pathologic N classification				
0	58 (60.4)	59 (67.8)	48 (42.9)	0.008
1	19 (19.8)	16 (18.4)	33 (29.5)	
2	19 (19.8)	12 (13.8)	31 (27.7)	

Values are presented as mean ± SD, number (%), or median (IQR)

*IQR *interquartile range, *EG * Esophagogastrostomy, *DTR *Double tract reconstruction, *TG* total gastrectomy with Roux-en-Y reconstruction, *BMI* body mass index, *ASA-PS* American Society of Anesthesiologists physical status

### Perioperative parameters

Table 2 shows the comparison of surgical outcomes in the DTR group with those in the EG and TG groups, respectively. The TG group exhibited a significantly shorter operation time (200(180,240) minutes vs. 230(210,255) minutes, *p* < 0.01) and a higher number of removed lymph nodes (28(22, 25) vs. 22(19.31)) when compared to the DTR group. However,, there were no significant differences in intraoperative blood loss, first postoperative exhaust time, postoperative hospital stay and postoperative complication rate among the three groups. The incidence of postoperative reflux symptoms was 9.2% in the DTR group, 43.8% in the EG group and 23.2% in the TG group. The DTR group had fewer reflux symptoms (*P* < 0.01).

**Table 2:** Comparison of surgical outcomes

	DTR (*n* = 87)	EG (*n* = 96)	*P* value
Operation time (min)	230 (210, 255)	195 (180, 250)	0.09
Bleeding (mL)	85 (50, 100)	100 (50, 100)	0.112
Number of retrieved lymph nodes	22 (19, 31)	21 (18, 26)	0.105
first postoperative exhaust time	3 (3, 5)	4 (3, 5)	0.067
Postoperative hospital stay (day)	7 (7, 9)	8 (7, 9)	0.732
Early complications (CD ≤ 2)	11 (12.6)	13 (13.5)	0.791
Early complications (CD = 3)	2 (2.3)	1 (1)	
Anastomotic leakage	1 (1)	1 (1)	
Anastomotic stenosis	1 (1)	0	
Gastrointestinal dysfunction	4 (4.5)	3 (3.1)	
Pulmonary infection	3 (3.4)	5 (5.2)	
gastrointestinal bleeding	1 (1)	0	
Intra-abdominal abscess	0	2 (2)	
Bowel obstruction	2 (2.2)	3 (3.1)	
Reflux esophagitis			
Yes	8 (9.2)	42 (43.8)	<0.001
NO	79 (90.8)	54 (56.3)	
	DTR (*n* = 87)	TG (*n* = 112)	*P* value
Operation time (min)	230 (210, 255)	200 (180, 240)	<0.01
Bleeding (mL)	85 (50, 100)	100 (50, 100)	0.538
Number of retrieved lymph nodes	22 (19, 31)	28 (22, 35)	0.001
first postoperative exhaust time	3 (3, 5)	4 (3, 5)	0.053
hospital stay(day)	7 (7, 9)	8 (7, 9)	0.628
Early complications (CD ≤ 2)	11 (12.6)	19 (17)	0.59
Early complications (CD = 3)	2 (2.3)	4 (3.6)	
Anastomotic leakage	1 (1)	3 (2.6)	
Anastomotic stenosis	1 (1)	4 (3.5)	
Gastrointestinal dysfunction	4 (4.5)	6 (5.4)	
Pulmonary infection	3 (3.4)	4 (3.5)	
gastrointestinal bleeding	1 (1)	2 (1.8)	
Intra-abdominal abscess	0	1 (0.9)	
Bowel obstruction	2 (2.2)	3 (2.6)	
Reflux esophagitis			
Yes	8 (9.2)	26 (23.2)	0.009
NO	79 (90.8)	86 (76.8)	

Values are presented as number (%), or median (IQR)

*IQR* interquartile range, *EG* Esophagogastrostomy, *DTR *Double tract reconstruction, *TG *total gastrectomy with Roux-en-Y reconstruction, Complications were defined according to the Clavien-Dindo classification system

### Double-tract reconstruction digestive tract radiography

**Fig. 4:**
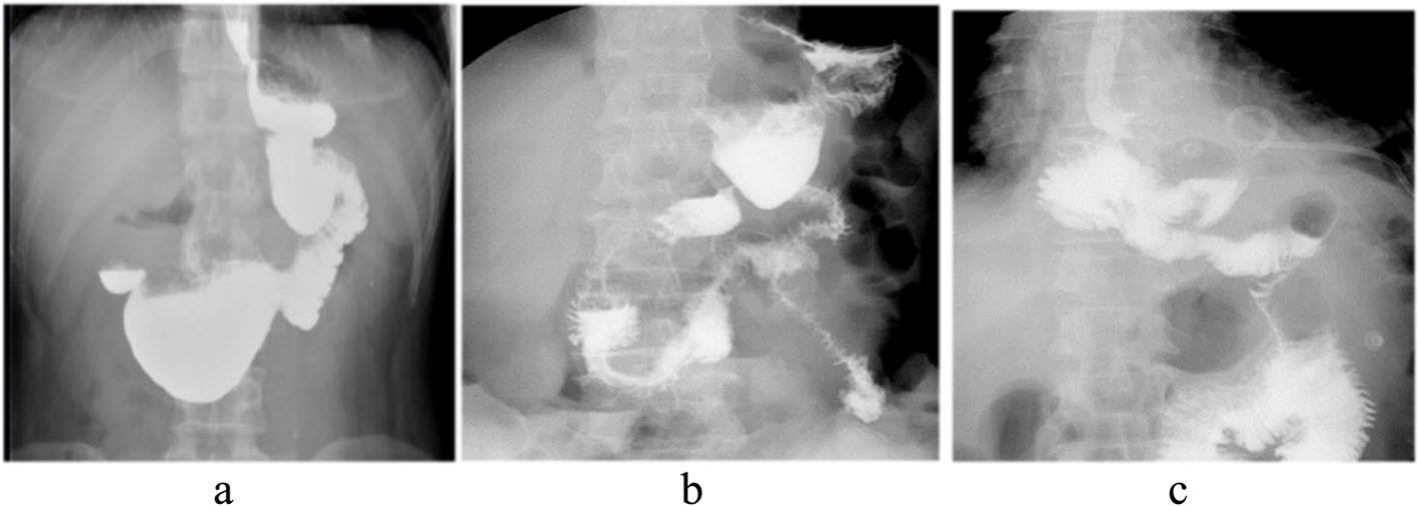
Representative postoperative fluoroscopic images. **a **the contrast medium passed only through the remnant stomach, (**b**) fluid passed through both the remnant stomach and the jejunum. **c** fluid passed only through the jejunum

A total of 36 patients with double-tract reconstruction completed postoperative gastrointestinal angiography, of which 21 (58.3%) showed double-channel and 15 (41.7%) showed changes after total gastrectomy. Figure 4 shows the results of the gastrointestinal radiography.

### Postoperative quality of life

**Fig. 5:**
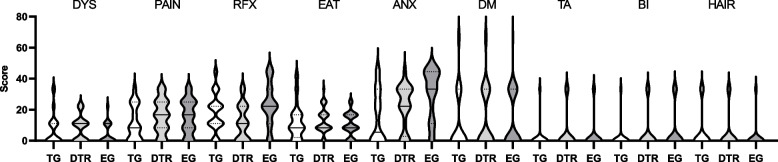
Violin plots of the symptom scales of the EORTC QLQ-STO22 questionnaire. Solid lines represent medians and dotted lines represent quartiles. A higher score represented worse symptoms. TG, total gastrectomy with Roux-en-Y reconstruction; DTR, Double tract reconstruction; EG, esophagogastrostomy; DYS, dysphagia; PAIN, pain; RFX, reflux; EAT, eating; ANX, anxiety; DM, dry mouth; TA, taste; BI, body image; HAIR, hair loss

Table 3 shows the EORTCQLQ-STO22 questionnaire survey 1 year after surgery. The EORTCQLQ-STO22 questionnaire survey showed that compared with EG group, DTR group had fewer reflux symptoms (*P* < 0.05), fewer anxiety symptoms (*P* < 0.05) and more swallowing symptoms (*P* < 0.05). There were no significant differences in pain, food restriction, anxiety, dry mouth, taste change, body image and hair loss (*P* > 0.05). Compared with TG group, the DTR group had fewer reflux symptoms (*P* < 0.01). There were no significant differences in anxiety symptoms, swallowing symptoms, pain, food restriction, anxiety, dry mouth, taste changes, body image and alopecia between two groups(*P* > 0.05). Figure 5 shows the Violin plots of the symptom scales of the EORTC QLQ-STO22 questionnaire.

**Table 3:** The scores of the EORTC QLQ-STO22 questionnaire

EORTC QLQ-STO22	DTR (*n* = 46)	TG (*n* = 79)	Z value	*P* value
Dysphagia	11.11 (0,11.11)	0 (0,11.11)	-0.86	0.39
Pain	16.67 (8.33,25)	8.33 (0,25)	-1.895	0.058
Reflux	16.67 (11.11,22.22)	11.1 (0,22.22)	-1.104	0.049
Eating	8.33 (8.33,16.67)	8.33 (0,16.67)	-0.988	0.323
Anxiety	22.22 (0,33.33)	11.11 (0,33.33)	-1.781	0.075
Dry mouth	0 (0,33.33)	0 (0,33.33)	-0.822	0.411
Taste	0 (0.0)	0 (0.0)	-1.331	0.183
Body image	0 (0.0)	0 (0.0)	-1.331	0.183
Hair loss	0 (0,0)	0 (0,33.33)	-0.198	0.843
EORTC QLQ-STO22	DTR (*n* = 46)	EG (*n* = 68)	Z value	*P* value
Dysphagia	11.1 (0,11.1)	0 (0,11.1)	-2.146	0.032
Pain	16.67 (8.33,25)	16.67 (8.33,25)	-0.148	0.883
Reflux	11.11 (0,22.22)	22.2 (11.1,33.3)	-3.431	0.001
Eating	8.33 (8.33,16.67)	8.33 (8.33,16.67)	-1.234	0.217
Anxiety	22.22 (0,33.33)	33.33 (11.11,44.44)	-2.632	0.008
Dry mouth	0 (0,33.33)	0 (0,33.33)	-0.085	0.932
Taste	0 (0.0)	0 (0.0)	-0.672	0.502
Body image	0 (0.0)	0 (0.0)	-0.301	0.763
Hair loss	0 (0,0	0 (0,0)	-1.055	0.292

Values are presented as median (IQR)

*IQR* interquartile range, *EG* Esophagogastrostomy, *DTR* Double tract reconstruction, *TG* total gastrectomy with Roux-en-Y reconstruction, *EORTC* European Organization for Research and Treatment of Cancer

### Nutritional status

**Fig. 6:**
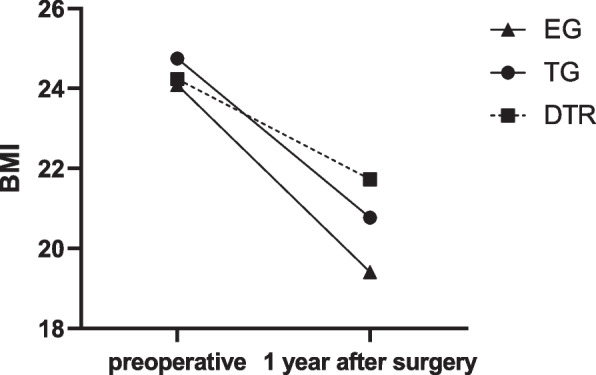
Comparison of postoperative changes in BMI between three groups. EG, Esophagogastrostomy; DTR, Double tract reconstruction; TG, total gastrectomy with Roux-en-Y reconstruction; BMI, body mass index

**Fig. 7:**
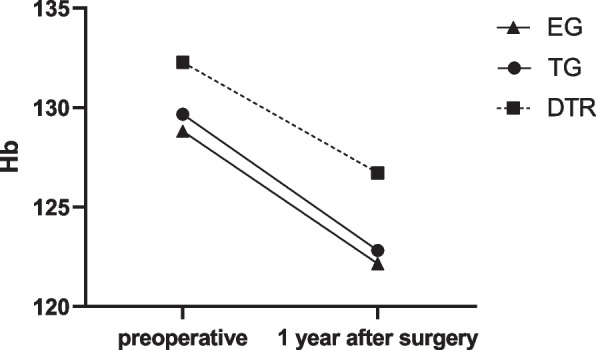
Comparison of postoperative changes in BMI between three groups. EG, Esophagogastrostomy; DTR, Double tract reconstruction; TG, total gastrectomy with Roux-en-Y reconstruction; Hb, hemoglobin

**Fig. 8:**
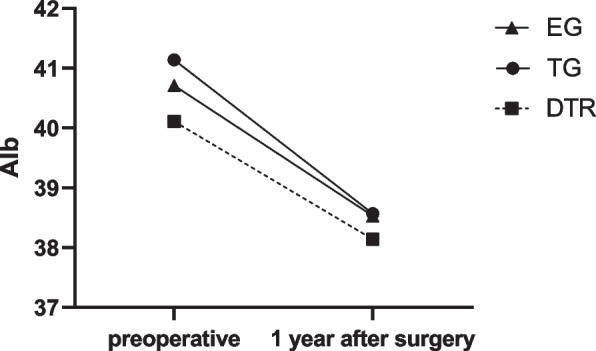
Comparison of postoperative changes in Alb between three groups. EG, Esophagogastrostomy; DTR, Double tract reconstruction; TG, total gastrectomy with Roux-en-Y reconstruction; Alb, Albumin

Nutritional parameter changes were evaluated, including BMI, hemoglobin and serum albumin, in three groups. Table 4; Fig. 6 shows The comparison of postoperative change in BMI between EG group 、DTR group and TG group. Compared with TG group and EG group, DTR can better maintain postoperative BMI. Table 5; Fig. 7 shows The comparison of postoperative change in Hb between EG group 、DTR group and TG group. Table 6; Fig. 8 shows The comparison of postoperative change in ALB between EG group, DTR group and TG group. and there is no statistical difference between the three groups in terms of hemoglobin and albumin.

**Table 4:** Comparison of postoperative change in BMI

BMI	DTR (*n*=64)	EG (*n*=79)	*P* value
preoperative	24.24 ± 3.27	23.79 ± 2.79	0.608
1 year after surgery	21.73 ± 2.90	19.42 ± 2.78	0.07
different	2.51 ± 1.38	4.37 ± 1.81	＜0.01
BMI	DTR (*n*=64)	TG (*n*=97)	*P* value
preoperative	24.24 ± 3.27	24.75 ± 3.36	0.611
1 year after surgery	21.73 ± 2.90	20.77 ± 3.17	0.286
different	2.51 ± 1.38	3.96 ± 1.59	0.02

Values are presented as mean±SD

*EG* Esophagogastrostomy, *DTR* Double tract reconstruction, *TG* total gastrectomy with Roux-en-Y reconstruction, *BMI* body mass index

**Table 5:** Comparison of postoperative change in Hb

Hb	DTR (*n* = 64)	EG (*n* = 79)	*P* value
preoperative	132.28 ± 19.20	128.82 ± 20.16	0.44
1 year after surgery	126.72 ± 10.76	122.15 ± 10.27	0.059
different	5.56 ± 14.09	6.67 ± 14.89	0.738
Hb	DTR (*n* = 64)	TG (*n* = 97)	*P* value
preoperative	132.28 ± 19.20	129.67 ± 18.19	0.539
1 year after surgery	126.72 ± 10.76	122.82 ± 8.48	0.08
different	5.56 ± 14.09	6.85 ± 14.67	0.695

Values are presented as mean ± SD

*EG* Esophagogastrostomy, *DTR* Double tract reconstruction, *TG* total gastrectomy with Roux-en-Y reconstruction, *Hb* Hemoglobin

**Table 6:** Comparison of postoperative change in ALB

Alb	DTR (*n*=64)	EG (*n*=79)	*P* value
preoperative	40.11 ± 3.21	40.71 ± 3.00	0.401
1 year after surgery	38.14 ± 3.90	38.53 ± 2.86	0.621
different	1.97 ± 3.66	2.18 ± 2.17	0.759
Alb	DTR (*n*=64)	TG (*n*=97)	*P* value
preoperative	40.11 ± 3.21	41.14 ± 2.76	0.135
1 year after surgery	38.14 ± 3.90	38.57 ± 2.93	0.592
different	1.97 ± 3.66	2.58 ± 2.69	0.409

Values are presented as mean±SD

*EG* Esophagogastrostomy, *DTR* Double tract reconstruction, *TG* total gastrectomy with Roux-en-Y reconstruction, *Alb* Albumin

## Discussion

In theory, double tract reconstruction is an ideal reconstruction method after proximal gastrectomy. Food can enter the distal digestive tract through two ways, which not only solves the problem of esophageal reflux after proximal gastrectomy, but also preserves the storage and digestive function of the residual stomach [9–11]. Most studies have shown that the anti-reflux effect of double tract reconstruction is exact [12, 13], but whether the quality of life and nutritional status of patients after double tract reconstruction are better than those of esophagogastrostomy and total gastrectomy with Roux-en-Y reconstruction is still controversial [15, 16].

Currently, the majority of studies have demonstrated that double tract reconstruction does not exhibit any significant differences in perioperative complications, in comparison to gastroesophageal anastomosis and total gastrectomy [4, 5, 17]. Nearly all studies have demonstrated that double tract reconstruction possesses a robust anti-reflux effect. Regarding postoperative quality of life and nutritional status, various studies have reported differing outcomes. s. Some studies have revealed that the quality of life and nutritional status after double tract reconstruction are superior to those after esophagogastrostomy and total gastrectomy [13, 18, 19], On the other hand, other studies have shown no significant differences in the quality of life and nutritional status after double tract reconstruction compared to esophagogastrostomy and total gastrectomy [20–22].

In this study, we compared clinical and nutritional outcomes and quality of life in the EG, DTR, and TG groups. We found that DTR had significant benefits in terms of nutritional outcomes as well as quality of life, especially for postoperative BMI maintenance.

The findings exhibit significant heterogeneity across studies, potentially attributable to the predominantly retrospective nature of the investigations and the arduousness associated with evaluating the quality of life scale. The assurance of both the quantity and quality of follow-up pertaining to the quality of life scale remains uncertain, while the comprehensive acquisition of nutritional assessment indicators at a precise time point poses challenges.

In 1988, Aikou et al. improved the traditional double-channel digestive tract reconstruction method by rotating the remnant stomach 180 degrees before gastrointestinal anastomosis, and restoring the remnant stomach to the normal position after anastomosis. The gastrojejunal anastomosis has an “N” shape, so that food can more easily enter the remnant stomach [23]. In laparoscopic-assisted application, the method of gastrointestinal anastomosis is simplified, and the linear stapler is used for anastomosis, although its anastomosis is wide enough, it may be closed in some cases [24].

Regarding the efficacy of double tract reconstruction, some studies have suggested that in some cases food cannot be emptied according to the theoretical double tract design, and some have suggested that when most food escapes directly through the jejunum, its function will be similar to total gastrectomy. Our investigation has revealed that following double tract reconstruction, two distinct states emerge: single tract and double tract. Gastrointestinal angiography showed that 58.3% of the patients presented double tract but DTR is superior to TG in reducing reflux symptoms and maintaining postoperative BMI. The presence of the remnant stomach preserves the secretion of gastric hormones and is beneficial to the balance of gastrointestinal hormones.

Body weight is an important indicator for postoperative nutritional evaluation, as it can intuitively reflect the patient’s nutritional status, while albumin/hemoglobin are easily affected by other factors. Studies have shown that lean body loss (LBL) 5% or more at 1 month after surgery is an independent sensitive risk factor for patients with stage 2 or 3 gastric cancer undergoing radical gastrectomy to continue adjuvant chemotherapy. The 6-month continuation rate was 91.7% in patients with a loss of less than 5%, and 66.3% in patients with a loss of 5% or more (*p* = 0.031). In addition, the LBL incidences of grade 3 toxicity to 5% or higher (42.9%) than LBL group 5% or less (18.9%) (*p*= 0.050) [25]. Our study shows that double-tract reconstruction can better maintain the postoperative weight of patients, on the one hand, it may be due to the retention of a part of the residual stomach is beneficial to the balance of gastrointestinal hormones and increase the absorption of nutrients. On the other hand, due to the low incidence of postoperative reflux symptoms in patients with double-tract reconstruction, our follow-up showed that the reflux rates of double-tract reconstruction, gastroesophageal anastomosis, and total gastrectomy were 9.2%, 43.8%, and 23.3%, respectively. the low incidence of postoperative reflux symptoms contributes to increased food intake. Although our study found that about 40% of patients did not form an effective double channel after double tract reconstruction, the double channel structure was still effective in reducing reflux, which indirectly indicates that double tract reconstruction can reduce not only acid reflux but also basic reflux.

Our study has several limitations that should be acknowledged. Firstly, the study was a retrospective study, which may indicate the presence of selection bias. Moreover, the number of patients in each group was relatively low, potentially limiting the generalizability of our findings. Secondly, the follow-up period was brief, and not all patients were available for follow-up, which could affect the accuracy and reliability of our results. Lastly, the nutrition indicators employed were not comprehensive, which may have led to an incomplete understanding of the patients’ nutritional status.

The quality of life and nutritional status after proximal gastrectomy is a significant research interest. However, there are several challenges in this area. Firstly, in retrospective studies, the follow-up success and accuracy rate of the life quality scale are typically low, which may affect the reliability and validity of the research findings. Secondly, the scale is too subjective, leading to wide variations in the evaluation of the same degree of discomfort among individuals of different ages, states of health, and mindsets. This variability is not attributable to differences in surgical methods, indicating that the current psychological scale is not entirely suitable for assessing the quality of life in post-surgical patients.

Therefore, there is a need for a simpler, more effective scale that can better evaluate the impact of surgery on patients’ lives. A scale that combines both subjective and objective evaluation methods would be ideal for this purpose, as it would enable more accurate assessments of patients’ quality of life post-surgery.

In conclusion, although the partial double-tract reconstruction approach does not always ensure food passage into the distal jejunum through both pathways as planned, the present analysis indicates that this technique appears to be an ideal method of anastomosis after proximal gastrectomy it has good anti-reflux function and can better maintain the BMI of patients after surgery. However, larger studies conducted over longer follow-up periods are needed to draw a more accurate comparison with other techniques.

## Data Availability

All publicly available data generated or analyzed during this study are included in this article. Further enquiries can be directed to the corresponding author.
